# Sprue
1Read before the Bath and Bristol Branch of the British Medical Association at Bath, February 22nd, 1905.


**Published:** 1905-06

**Authors:** Charles Begg


### SPRUE.[1]

BY

CHARLES BEGG, M.B., C.M. Edin.

---

A DISEASE of tropical countries, characterised by continued diarrhœa, large, bulky, pale-coloured motions out of all proportion to the food taken, with resulting emaciation caused by an invasion of the intestine by micro-organisms, resulting in chronic ill-health, death by exhaustion, or by intercurrent disease. Such shortly is the disease called sprue.

In 1882 I had been three years in China without the knowledge that such a disease existed. A reprint then reached me from a London physician detailing six cases treated by milk, and the faulty pathology given first attracted my attention. It was described as a primary atrophy of the mucous membrane from mouth to anus. I had been taught that atrophy must always be a secondary process. I then was struck by the similarity of the clinical symptoms described with those of several cases that had occurred during my three years' practice—cases I had failed to cure by any known method of treatment. The obviously faulty pathology stimulated me to attack the problem afresh, so I looked up my old cases and found them still suffering. On an extra-careful examination I was struck with the prominent fact that the tongue was markedly clean, even irritable looking, and in order to eliminate the presence of lumbrici I put them on doses of the old-fashioned yellow santonine, telling them to keep a watch on the motions for the passage of a worm. We never saw any, but the diarrhœa ceased and normal health returned. Failing a macroscopic, I had to fall back on the theory of a microscopic invasion of the bowel, and after a number of successful cases I published my results.

[1] Read before the Bath and Bristol Branch of the British Medical Association at Bath, February 22nd, 1905.

On a visit to England in 1890, after adding to my experience certain cases I got into touch with in London and which had been pronounced incurable, I read a paper before the Edinburgh Medico-Chirurgical Society, which was published in the *Edinburgh Medical Journal* for September of that year. I had worked out a probable pathology which would explain all the clinical facts, and I had the satisfaction of finding this to be demonstrated to be correct by F. J. Wethered, from slides taken from a patient who died in London during the same year. By this time I had made the interesting discovery that it was not the santonine that cured, but a by-product formed by the action of light on the white drug; and success depended on the amount of such decomposition, the white santonine now supplied by chemists being absolutely useless. So far I have failed to have this substance identified.

Such is my connection with this disease in the past, and I intend now to give very briefly a clinical picture of it, relate some recent cases, and say something about the difficulties of treatment when one is dealing with the chronic cases seen in this country.

The disease as a rule begins insidiously, two or three motions in the morning and then free for that day. The motions are very bulky, of a light clay colour, sometimes quite white, passed generally without pain or urgency, frequently in a state of apparent fermentation, being often covered with air bubbles, reaction acid instead of alkaline. They contain no undigested material, and are largely in excess of the food taken. There is steady, often rapid, loss of weight, and a most uncomfortable sensation referred to the abdomen. Flatulency, with painful distension, is a common symptom. Almost always there is tenderness or even pain in the mouth if hot fluids or spiced foods be taken. At times the tongue becomes raw and sore, but is never foul or furred. There is no jaundice or bile in the urine, which is pale and of low specific gravity. The liver is generally smaller than natural, often markedly so. Patients look anxious and depressed. There is no fever, and the pulse is slow and weak. When the case becomes chronic, there is still the marked tendency for the diarrhœa to be only in the morning, and with the profound emaciation there comes a more or less marked condition of anæmia. All the symptoms vary from time to time. In advanced cases the tongue is glazed, shiny, very red, and raw looking.

On examination of such a patient, after noting the emaciation, the peculiar condition of the tongue, the absence of temperature, the shrunken condition of the liver and appearance of serious illness, you cannot fail to notice the unexpected appearance of the abdomen. It often shows up large and tumid by contrast to the wasted limbs. It has a peculiar soft, doughy feeling on palpation. There may be slight tenderness on pressure, and as a rule the note is tympanitic. The motions are very characteristic. From a consideration of the clinical facts and the results of treatment, I came to the conclusion that we had to deal with a micro-organism which in some way confined itself to rendering the products of digestion incapable of being absorbed, and that therefore we had to do with a patient who was being starved. I pointed out reasons for believing that the gastric mucous membrane did not share in the process, that the lower bowel was as a rule also free, and that the mucous membrane of the mouth was only affected in a secondary way as a result of the malnutrition, much as we see in other wasting diseases.

The results of the *post mortem* which cleared up these points were published after I read my paper, and I had an opportunity of going over the slides with Dr. Wethered, when he agreed that they established my view of the disease. Very shortly, what he found was as follows. Tongue, epithelium in some places appears to be absent. Œsophagus, practically no change. Stomach, no change, except that its surface had some of the mucoid-like material which was found so largely in the gut below. The intestines had a velvety feel as if thickened, but the walls of duodenum, jejunum and ileum were found to be thin, while the surface was lined by a thick layer of mucoid-like material, which could be scraped off, leaving the gut wall in parts thin and translucent. The large intestine also showed similar changes, but not so marked. Under the microscope the greatest changes were seen in the ileum. The mucosa was almost entirely destroyed, being replaced by a structureless substance enclosing leucocytes and remains of the glandular substance. The submucosa thickened, fibrous tissue abundant. The layer of mucoid-like material was studded with rod-shaped bodies; portions of this are sometimes passed by patients and are seen to present the same appearances. Stomach, liver and pancreas normal, hence digestion perfect, but the absorbing surface of the intestine first shut off, then destroyed by the pressure and irritation of the shutting-off material, hence patients with perfect digestions and good appetites are starved from failure to absorb the products of digestion. Treatment we may divide into two. We have first to kill the micro-organism, and in the second place to regulate the function of the bowel.

In a recent case neither process gives much trouble, but in the class of cases met with in this country—cases of years' standing—the problem is a much more complex one. Experience has taught me many lessons as to the best way to give the remedy—which is santonine—and to prepare the patient to obtain its full action. But the remedy itself must be of the right kind. It is not enough to be of a pale yellow colour; the longer it is exposed to light the better and the darker it becomes. I have some coming soon from India, where it has had a period of exposure to strong sunlight. I have experimented with the X-rays to a slight extent, but have not found it act better than ordinary sunlight. I give the remedy in oil to carry it past the stomach. I tried keratine-coated capsules, but they were unsatisfactory. Experience will guide you as to the necessity of clearing the bowels before administration, and in the number of doses required before you pause to see if it has done its work, and if you can now proceed to regulate the function. You can tell this by the state of the tongue, when you will observe a delicate fur appear over the bare patches, and the patient will be passing less bulky motions, perhaps formed, and certainly with approach to the natural brown colour. At this stage B. naphthol, salol, &c., are useful, as also is opium in some form to delay the motions in the bowel to allow of part being absorbed; it checks the accustomed hurry through the gut. Various bitters are useful before meals, as well as certain preparations of iron. In both stages diet has to be regulated, no two cases being alike. All can digest any kind of food as far as sprue goes, but certain foods seem to cause the resulting product to be hurried through. All the fats and oils come under this head—bacon, butter, fat meat, &c. In the same way the quantity of any liquid taken must be kept small for each dose. In certain cases it is essential to keep the patient on a strict milk diet and in bed, in order to allow the santonine to remain as long as possible in the gut. Sometimes the milk must be evaporated. Others do well on meat juice, fish, stale bread, and underdone steak. One of my patients at present under treatment has been allowed from the first to eat everything, and is fast gaining weight and strength. Another requires the most careful diet, and appears to do best on pure milk.

Dr. Bullmore has been good enough to undertake a research into the blood condition in this disease. When a sufficient number of observations have been made the results will be published. In the meantime, he allows me to say that the appearances are the same as that found in a case of pure malnutrition. So far four cases have been observed, and in them the average count has been: Red corpuscles, 4,170,000; hæmoglobin, 76 per cent.; white corpuscles, 5,780. In two of the cases slight polychromatophilia and poikilocytosis were apparent. An average differential count of the white corpuscles gives:

| | | |
|---|---|---|
| Polymorphs ... ... ... ... ... ... ... | 53·5 | per cent. |
| Lymphocytes ... ... ... ... ... ... ... | 33·1 | ,, |
| Large mononuclears or transitionals... ... | 8.95 | ,, |
| Eosinophiles ... ... ... ... ... ... ... | 2.16 | ,, |
| Basophiles ... ... ... ... ... ... ... | 0.88 | ,, |

Thus showing a relative and absolute decrease in the polymorphs, a relative increase in lymphocytes, and a relative and actual increase in the large mononuclears. No glycogenic reaction was found. In one case examination during the administration of santonine showed a marked improvement, being a return to normal conditions. The hæmoglobin had risen from 70 per cent. to 95 per cent., the red corpuscles remained about the same, the white corpuscles had risen from 5,400 to 6,200.

| | Examined Dec. 14th, 1904. | Feb. 2nd, 1905. |
|---|---|---|
| Polymorphs ... ... | 47.5 per cent. | 59.69 per cent. |
| Lymphocytes ... ... | 36.5 ,, | 31.46 ,, |
| Large mononuclear | 10.04 ,, | 6.63 ,, |
| Eosinophiles ... ... | 3.03 ,, | 1.3 ,, |
| Basophiles ... ... | 1.3 ,, | 1.2 ,, |

In conclusion, I would point to the many interesting problems introduced by this disease and its treatment. The micro-organism found in the mucoid-like material covering the free surface of the bowel, what is it? How is the material in which it is found produced? and from where? *Post mortems* are rare, and when obtained are only in very severe cases or in those who have died from intercurrent disease; but from what we have seen it is a fair inference that in ordinary recent cases the mucous membrane is little if at all damaged, and if we can get rid of the covering which prevents the properly digested nourishment reaching the absorbing surface the patient recovers absolutely. This takes place on the death of the micro-organism. Why are the motions light-coloured—often absolutely white? What becomes of the bile and its colouring matter? Does it fail to be secreted in sufficient quantity by the liver in its shrunken condition? or is it decolourised in the intestine by some chemical process? What is the by-product formed by the action of light on santonine? Similar changes are known in other drugs, and they are held to change their character little if at all. That santonine is changed I have the strongest of clinical proof, having over and over again demonstrated the failure of the white and curative power of the yellow in the same patient.

In the time at my disposal I have had to stick to the broad characteristics of the disease, but I trust I have said enough, and brought forward enough cases in proof of my contention as to the curability of this disease, to give you an additional interest in any case that may come under your notice, and induce you to give a trial to impure santonine, and not to adopt the present attitude of the profession towards the disease, condemning such patients to a life of bed and milk and prohibiting a return to the country where they were infected.

